# Microbiomes of a specialist caterpillar are consistent across different habitats but also resemble the local soil microbial communities

**DOI:** 10.1186/s42523-020-00055-3

**Published:** 2020-10-07

**Authors:** Sofia I. F. Gomes, Anna M. Kielak, S. Emilia Hannula, Robin Heinen, Renske Jongen, Ivor Keesmaat, Jonathan R. De Long, T. Martijn Bezemer

**Affiliations:** 1 Department of Terrestrial Ecology, The Netherlands Institute of Ecology (NIOO-KNAW), Droevendaalsesteeg 10, 6708 PB Wageningen, The Netherlands; 2 Present Address: Lehrstuhl für Terrestrische Ökologie, Wissenschaftszentrum Weihenstephan für Ernährung, Landnutzung und Umwelt, Technische Universität München, Hans-Carl-von-Carlowitz-Platz 2, 85354 Freising, Germany; 3 Present Address: Greenhouse Horticulture, Wageningen University and Research, Violierenweg 1, 2665 MV Bleiswijk, The Netherlands; 4 Institute of Biology, Section Plant Ecology and Phytochemistry, Leiden University, P.O. Box 9505, 2300 RA Leiden, The Netherlands

**Keywords:** Insects, Lepidoptera, *Ralstonia*, Soil, *Tyria jacobaeae*, *Jacobaea vulgaris*, Insect populations

## Abstract

**Background:**

Insect-associated microorganisms can provide a wide range of benefits to their host, but insect dependency on these microbes varies greatly. The origin and functionality of insect microbiomes is not well understood. Many caterpillars can harbor symbionts in their gut that impact host metabolism, nutrient uptake and pathogen protection. Despite our lack of knowledge on the ecological factors driving microbiome assemblages of wild caterpillars, they seem to be highly variable and influenced by diet and environment. Several recent studies have shown that shoot-feeding caterpillars acquire part of their microbiome from the soil. Here, we examine microbiomes of a monophagous caterpillar (*Tyria jacobaeae*) collected from their natural host plant (*Jacobaea vulgaris*) growing in three different environments: coastal dunes, natural inland grasslands and riverine grasslands, and compare the bacterial communities of the wild caterpillars to those of soil samples collected from underneath each of the host plants from which the caterpillars were collected.

**Results:**

The microbiomes of the caterpillars were dominated by Proteobacteria, Actinobacteria, Firmicutes and Bacteroidetes. Only 5% of the total bacterial diversity represented 86.2% of the total caterpillar’s microbiome. Interestingly, we found a high consistency of dominant bacteria within the family Burkholderiaceae in all caterpillar samples across the three habitats. There was one amplicon sequence variant belonging to the genus *Ralstonia* that represented on average 53% of total community composition across all caterpillars. On average, one quarter of the caterpillar microbiome was shared with the soil.

**Conclusions:**

We found that the monophagous caterpillars collected from fields located more than 100 km apart were all dominated by a single *Ralstonia*. The remainder of the bacterial communities that were present resembled the local microbial communities in the soil in which the host plant was growing. Our findings provide an example of a caterpillar that has just a few key associated bacteria, but that also contains a community of low abundant bacteria characteristic of soil communities.

## Background

Insects are associated with diverse microorganisms [1], and most well studied microbial diversity in insect guts comprises of bacteria [2]. Interactions between insects and their associated bacterial communities range from mutualistic to pathogenic. A few of these symbiotic interactions have been intensively studied [3], suggesting that symbiotic microbiota provide nutrients and improve insect digestion [4, 5]. In caterpillars, microbiomes also play important roles in protecting the insects against pathogens [6]. Yet, the low degree of morphological specialization in the gut and the rapid transit of food through the digestive tracks of caterpillars suggest a minor role for the microbial communities in their physiology [7]. These opposite trends emphasize the need to examine caterpillar-associated microbial communities. Furthermore, most of our understanding of caterpillar microbiomes stems from caterpillars cultured under laboratory conditions and there is a dearth of studies that examine caterpillar-microbe interactions in their natural habitats.

A recent study finds that caterpillars lack a resident gut microbiome and that the bacteria found inside the insect guts are transient [8]. The origin of these variable microbial communities inside caterpillars is not well-understood, as gut microbiomes do not always reflect the bacterial composition present in the diet or in the local environment [2, 9]. Despite their transient nature, bacterial assemblages in insect guts do not seem to be random associations [2, 10]. Insects collected in several localities can have highly variable microbial communities partially shaped by the host plant (e.g. [4]). For example, the microbiomes of larvae of the gypsy moth and the cabbage white butterfly are dependent on the diet but also on the presence of widespread environmental taxa [11, 12]. Furthermore, a recent study showed that cabbage moth caterpillars acquire their microbiomes largely from the soil rather than from the host plant they feed on [13]. This leads to the hypothesis that the microbial communities in the soil may directly shape the variable microbiomes of caterpillars.

The caterpillars of the cinnabar moth (*Tyria jacobaeae* (L.); Lepidoptera: Arctiidae) are monophagous herbivores of the common ragwort (*Jacobaea vulgaris* (Gaertn.); Asteraceae) [14] and are used as biological control of this weed that is invasive in multiple continents [15]. These caterpillars are able to circumvent the potentially harmful effects of the high levels of pyrrolozidine alkaloids contained in ragwort plants [16]. The activity of specific gut associated bacteria may contribute to such adaptation to toxic components, as observed in other phytophagous insect species [3, 4, 17]. Caterpillar microbiomes generally vary among individuals [8], and the cinnabar moth caterpillar is monophagous [14], thus diet likely plays a restricted role in shaping the variable microbial communities, contributing little to explain the beta diversity (i.e. variation among individuals). We therefore hypothesize that this caterpillar species may contain a small core microbiome, and that the remaining bacteria that make up the total microbiome will reflect the soil microbiome of their habitat.

To investigate the effect of soil microbial communities on the microbiome of wild populations of these caterpillars at the landscape level, we sampled caterpillars of the cinnabar moth and soil in nine localities across three habitats in the Netherlands – coastal dunes, inland natural grasslands and riverine grasslands – which due to their characteristic soil properties, harbor distinct bacterial communities [18]. The soil and caterpillar associated bacterial communities were characterized by sequencing the 16S rRNA gene.

## Methods

### Study sites and sample collection

We selected three characteristic habitats in The Netherlands in which ragwort plants and cinnabar moths occur in their native range: coastal dunes (Meijendel), inland natural grasslands (Veluwe) and riverine grasslands (Wageningen) and sampled in three localities within each habitat. At each locality, ten *Jacobaea vulgaris* plants on which *Tyria jacobaeae* caterpillars were feeding were selected (SI Figure S1). Plants were located at a distance of at least 10 m to each other (except for two sampling localities in riverine grasslands where the smallest distance between two plants was 8.3 and 9.2 m). Around the stem of each plant, five soil samples from the top 5 cm layer were taken using a 20 cm soil borer with 2 cm diameter and pooled together. Caterpillars collected from each individual plant were kept together (with a minimum of 3 and a maximum of 10 caterpillars collected per plant). All samples were stored in a cooler with ice until processing in the laboratory on the same day. Fresh weight of each individual caterpillar was recorded (SI Figure S2; SI Table S1). All caterpillars were surface sterilized by dipping them for 30 s in the following solutions: 70% ethanol, 2.0% bleach, and then rinsed twice with autoclaved demineralized water. Caterpillars were surface sterilized to enrich the samples for gut rather than surface microbes while leaving gut microbes intact [19]. Both caterpillar and soil samples were stored at − 20 °C until further processing. Caterpillar samples were lyophilized prior to DNA extractions.

### DNA extraction and library preparation

To obtain a representative sample of caterpillars feeding on one plant, DNA was extracted collectively from three homogenized caterpillars from approximately 10 mg of dry, lyophilized sample. In total, 270 caterpillars were used in this study (9 study locations × 10 plants × 3 caterpillars per sample). Extractions were performed using the MP Biomedicals FastDNA™ Spin Kit (MP Biomedicals, Solon, Ohio, USA) following the manufacturer’s protocol with the following modifications. Samples homogenized with Cell Lysis Solution in the FastPrep® Instrument (MP Biomedicals) for 20 s (speed setting of 6.0) were incubated at room temperature for 1 h. An extra washing step was included, and the final eluted DNA was additionally precipitated prior further purification using standard ethanol precipitation method with potassium acetate.

From approximately 0.35 g of soil samples, DNA was extracted from 90 samples using DNeasy PowerSoil Kit DNA (Qiagen, Hilden, Germany) following the manufacturer’s protocol.

Approximately 10 ng of template DNA was used for PCR using primers 515FB [20] and 806RB [21] targeting the V4 habitat of the 16S rRNA gene [22]. The PCR mixture (25 μl) contained 12.5 μl Phusion Flash High Fidelity PCR Master Mix (Thermo Scientific), 1.25 μl of each of the primers (10 μM). The conditions were 45 s at 98 °C, followed by 30 cycles for caterpillars and 25 for soil samples of 98 °C for 5 s, 55 °C for 5 s, and 72 °C for 10 s with a final extension of 1 min at 72 °C. The PCR products were purified using Agencourt AMPure XP magnetic beads (Beckman Coulter, Brea, CA, USA). Adapters and barcodes were added to samples using Nextera XT DNA library preparation kit sets A-B (Illumina, San Diego, CA, USA). The final PCR product was purified again with AMPure beads, verified using agarose gel electrophoresis and quantified with a Nanodrop spectrophotometer (Thermo Scientific, Hudson, NH, USA) before equimolar pooling. Separate libraries were prepared for bacterial communities derived from caterpillar and soil samples (96 samples per library) including extraction negatives. Libraries were sequenced at McGill University and Genome Quebec Innovation Center.

### Sequence processing

Raw reads were processed into ASVs (amplicon sequence variants) using the DADA2 pipeline [23], and taxonomic identification was performed by querying against the SILVA database with SINA classifier [24]. Reads that could not be assigned to Bacteria (i.e. Archaea, Eukaryotes, mitochondria, chloroplast, and unidentified) were excluded. Because the microbial abundance of caterpillars is often low [8], and this is one of the few studies to survey the bacterial communities from multiple individuals of *T. jacobaeae* collected in the field, it is challenging to distinguish true contaminants in the samples [25], therefore we removed the ASVs identified as *Cutibacterium* spp. as they were present in the negative control, and are usually human specific. However, we kept 14 ASVs that were also present in the negative controls. “These represented less than 10% of all reads in caterpillar samples, with the exception of two ASVs identified as Peptoniphilus and Dolosigranulum that have been described to be gut residents.” The percentage of mitochondria and chloroplast reads was variable between samples, as it is often reported in insect gut microbiome studies [26], but not significantly different between localities or regions (SI Figure S2). Caterpillar and soil datasets were filtered separately due to their inherent differences in bacterial diversity. Only samples with sequencing depth ranging from five times more to five times less than the mean sequencing depth of caterpillar or soil samples were kept for further analysis. To explore general patterns of bacterial diversity among samples, the caterpillar and soil datasets were resampled to the lowest sequencing depth of 3849 and 13,731 reads, respectively. This resulted in removing three caterpillar samples (one from each habitat); while all soil samples were kept. The combined rarefied datasets resulted in 1,570,653 reads assigned to 41,089 bacterial ASVs, of which 11,685 ASVs were present in high abundance (> 0.1% relative abundance).

### Microbiome analysis

Alpha diversity for caterpillar and soil was assessed on the rarefied datasets by calculating the estimated species richness, Shannon diversity (the exponential of Shannon entropy), and Simpson diversity (the inverse Simpson concentration) [27], using the *iNEXT* R package [28]. These indices are based on sample-size interpolation and extrapolation sampling curves [29], and represent the diversity estimates for rarefied and extrapolated samples with respect to the number of samples within localities. The estimated diversity was compared between habitats using the nonparametric Wilcoxon test with pairwise adjusted (Holm) *p*-values. Because caterpillars in the natural populations varied in size (see SI Table S1 and Figure S3 for average weights), to assess potential associations between the fresh biomass of caterpillars and their microbial species diversity or community composition, we used the Spearman rank correlation coefficient, and multivariate GLMs (see method description below), respectively. Moreover, to test whether spatial distribution of the sampled localities had an effect on the structure of bacterial communities in caterpillars, we computed a Mantel test correlation between the distance among localities in kilometers, calculated with the *geosphere* R package [30] on the geographic coordinates, and the Bray-Curtis dissimilarity matrix bacterial composition of caterpillars. The rarefied relative abundance data were squared-root transformed and Wisconsin double-standardized before the calculation of the Bray-Curtis dissimilarity matrix using the *vegan* R package [31]. This standardization scales the variability of different samples to each other.

To display differences in microbiome composition of caterpillar and soil samples among localities within the three habitats, we generated Principal Coordinates Analysis (PCoA) ordination plots using the *ape* R package [32], using the squared-root transformed and Wisconsin double-standardized data. To reduce the influence of taxa present in few samples to the overall community composition, only ASVs present in at least three caterpillar or soil samples with a relative abundance higher than 0.1% in each dataset were included in the PCoA. The homogeneity of dispersion of the microbiome data was tested using *betadisper* function in *vegan* R package, and for both caterpillars and soil in the nine localities within the three habitats, data were overdispersed. We recognize that distance-based approaches can perform poorly when the data presents strong mean-variance relationships [33], therefore we used it only as a means to visualize differences in microbiomes.

To assess caterpillar and soil microbiome community differences between localities and within habitats, we used a multivariate model-based approach [34], using the *mvabund* R package [35]. The microbiome structure of caterpillars or soil within locations or habitats was tested in separate models. We used a GLM with negative binomial distribution and a log-link function to account for the overdispersion of the data. In the multivariate GLM, a model was fit to each ASV and the log-likelihood ratio (LR) of each model was summed to create an overall sum-of-LR [34]. Examination of the residual plots from the models showed no clear patterns indicating that the negative binomial GLM model was appropriate. Significance of the models was evaluated using 999 resampling iterations with PIT-trap resampling [36]. In addition, a linear discriminant analysis coupled with effect size measurements (LEfSe) based on Wilcoxon sum-rank test [37] was used to screen for differentially abundant bacteria at any taxonomic level among the three habitats.

## Results

### Microbial diversity

**Fig. 1:**
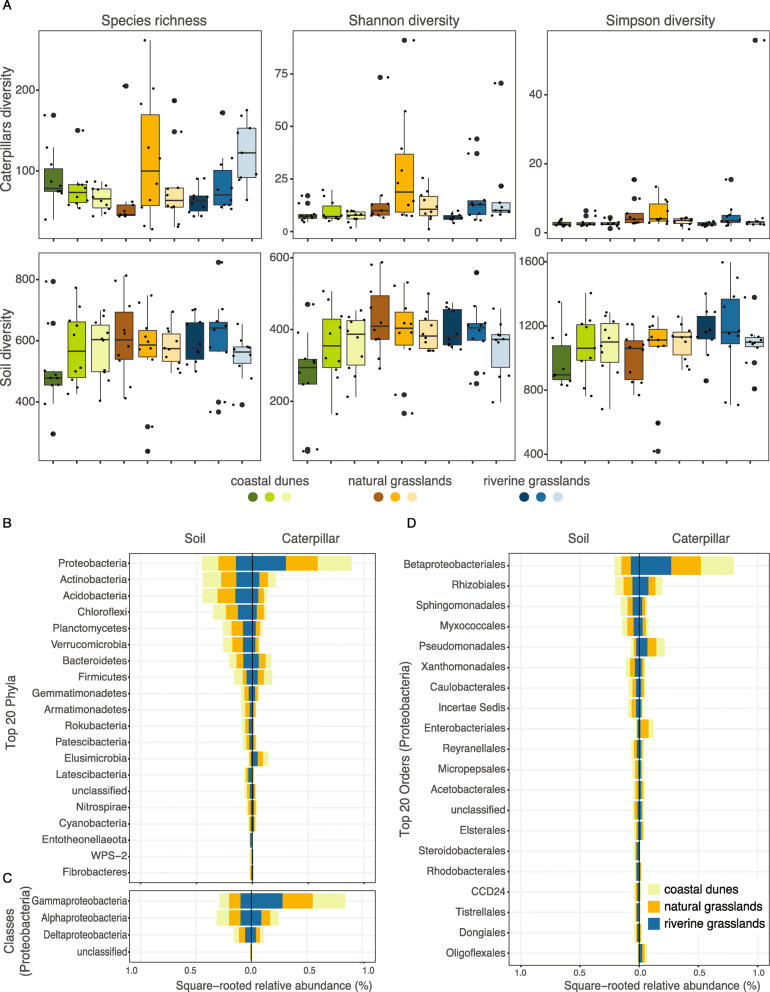
Alpha diversity of caterpillar and soil samples collected in the nine localities within three habitats (coastal dunes, natural grasslands and riverine grasslands). Species richness, Shannon diversity and Simpson diversity in caterpillar and soil microbiomes were calculated for the rarefied datasets. Wilcoxon tests with pairwise adjusted (Holm) *p-values* showed that diversity indices were not significantly different between localities or habitats (**a**). Overall abundances of bacterial top 20 phyla (**b**), classes (**c**) and top 20 orders (**d**) within the most abundant phylum (Proteobacteria) in caterpillar (right hand side) and soil (left hand side) samples. In (**b** to **d**) to improve visualization of lower abundant taxa, total relative abundance for each taxon was square-rooted transformed. The relative abundances of bacteria from each habitat is represented as proportional to the whole bar

We found 3080 (335,283 reads) bacterial ASVs unique to caterpillar and 9594 (1,136,338 reads) ASVs unique to soil samples out of the total 11,658 highly abundant ASVs (> 0.1% relative abundance in each dataset). At the selected sequencing depth, caterpillar rarefaction curves reached a plateau (SI Figure S4) suggesting that we sequenced most of the bacterial diversity in the caterpillars, which is further supported by the similar observed and estimated diversity indices (SI Table S1). However, soil rarefaction curves did not reach a plateau and estimated diversity indices were often greater than observed values (SI Figure S4 and SI Table S2), suggesting that we failed to obtain a complete representation of the soil bacteria. Pairwise comparisons of the diversity indices between localities or habitats showed no significant differences for both caterpillar and soil datasets (Fig. 1a). Moreover, caterpillar weight was neither related to the bacterial diversity indices (species richness: *ρ* = 0.09, *p* = 0.416; Shannon diversity: *ρ* = − 0.15, *p* = 0.160; Simpson diversity: *ρ* = − 0.21, *p* = 0.057; SI Figure S5), nor to community composition (multivariate GLM: *df* = 85, *deviance* = 4327, *p* = 0.059; SI Figure S6). Besides, the bacterial community structure of caterpillars was not spatially structured (Mantel statistics *r* = − 0.02, *p* = 0.778).

In caterpillar samples, Proteobacteria was the most abundant bacterial phylum constituting on average 78.8% reads of the total microbiome across habitats, followed by Actinobacteria (4.4%), Firmicutes (3.4%) and Bacteroidetes (2.8%) (Fig. 1b). Within the most abundant phyla, Gammaproteobacteria were the most abundant class (Fig. 1c) and Betaproteobacteriales the most abundant order (Fig. 1d). In soil samples, the most abundant phyla were Proteobacteria (20.6%), Actinobacteria (20.2%), Acidobacteria (19.5%), Chloroflexi (12.6%), Planctomycetes (6.9%), Verrrucomicrobia (6.8%), Bacteroidetes (4.4%) and Firmicutes (3.2%) (Fig. 1b).

### Caterpillar microbiome

**Fig. 2:**
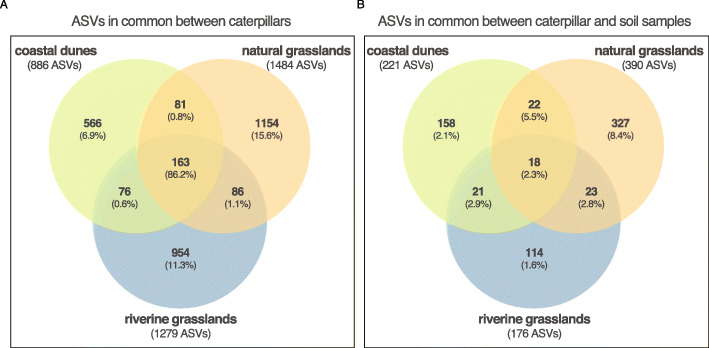
Venn diagrams illustrating overlap of ASVs across the three habitats. Caterpillars in the three habitats shared 163 ASVs of the total 3080 ASVs (**a**). Caterpillar and soil samples shared 683 ASVs within the same habitat which represents 25.6% of the caterpillar microbiome, and 18 ASVs were found in both caterpillars and soils across the three habitats representing 2.3% of their mean relative abundance (**b**). Numbers inside the circles in bold represent the number of ASVs in each particular habitat; numbers below, in between brackets, represent the mean relative abundance of those ASVs within the respective habitats

**Fig. 3:**
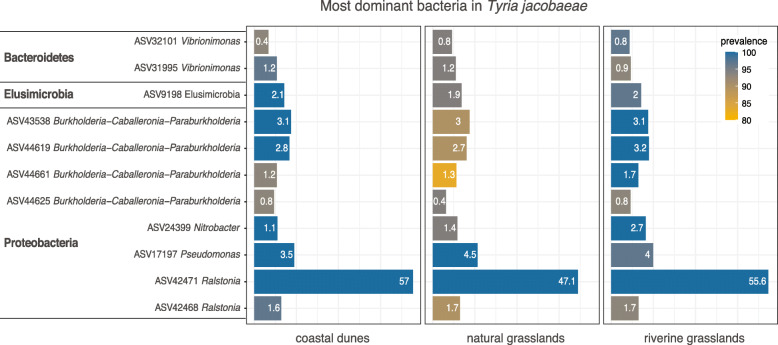
Most dominant bacterial ASVs detected in caterpillars. These 11 ASVs were found in at least 80% of all caterpillars within the nine localities across the three habitats. Mean relative abundance of bacteria in the locations within each habitat is depicted inside the bars. See SI Table S3 for overall mean prevalence and relative abundance

Of the 3080 ASVs present in *T. jacobaeae*, 886, 1484 and 1279 ASVs were detected in coastal dunes, natural inland grasslands and riverine grasslands, respectively. Across habitats, caterpillars shared 163 ASVs which accounted for 86.2% of the relative abundance of their total microbiomes, while most ASVs (64–78%) were characteristic to particular habitats representing 6.9–15.6% mean relative abundance of their microbiomes (Fig. 2a). We found the majority of caterpillar samples (> 80%) to share 11 bacterial ASVs from the phyla Proteobacteria, Bacteroidetes and Elusimicrobia (Fig. 3), of which 6 belonging to the genera *Ralstonia*, *Pseudomonas* and the complex *Burkholderia-Caballeronia-Paraburkholderia* were present in more than 90% of samples (SI Table S3). One *Ralstonia* ASV was detected in all caterpillars representing a mean of 53% of their entire microbiome. Of the 11 ASVs shared among most caterpillars, 10 were not present in any soil sample even before rarefying the soil dataset (*Nitrobacter* was the only taxon present in soil).

### Community composition in soils and in caterpillars

**Fig. 4:**
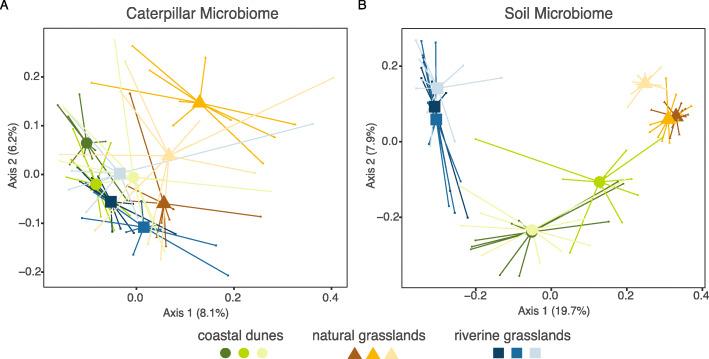
Microbiome community composition of caterpillars (**a**) and soil (**b**) represented by Principal Coordinates Analysis calculated with Bray-Curtis dissimilarities, with ASVs present in at least three samples and being highly abundant (≥ 0.1%). Percentage of variance explained by the axes is indicated between brackets

The removal of ASVs present in less than three samples for each dataset resulted in 215 ASVs (291,763 reads) in caterpillar and 4384 ASVs (982,414 reads) in soil datasets. Bacterial community composition was significantly different for both caterpillars and soil according to habitat and locality using the multivariate GLMs (Table 1). Pairwise comparisons of caterpillar and soil microbiomes indicated for both significant differences between the three habitats (Table 2). No significant differences were observed between any of the individual localities in caterpillars (SI Table S4) whereas soil microbiomes differed significantly between most of the localities (except no differences were found within the riverine grassland sites and two of the coastal dunes sites; SI Table S4). Consistent with these results, we observed differences in the bacterial community structure in caterpillars according to habitat (Fig. 4a), and more strongly in the soil samples (Fig. 4b). The LEfSe analysis showed no significant association with the three habitats or the nine localities of any bacteria taxa at any taxonomic level.

**Table 1:** Microbial composition of caterpillars and soil samples

	Variable	Res. Df	Deviance	Adj. ***p***-value
**Caterpillars**	Intercept	86		
	Habitat	84	640.9	**0.001**
	Intercept	86		
	Locality	78	2317	**0.005**
**Soil**	Intercept	89		
	Habitat	87	50,605	**0.001**
	Intercept	89		
	Locality	81	83,275	**0.005**

A negative binomial GLM was used. Multivariate test statistics were calculated using the log-likelihood ratio with 999 iterations via PIT-trap resampling. Significant results (*p-value* < 0.05) are in bold

Analysis of deviance table for bacterial community composition of caterpillars and soil samples in the nine localities within the three habitats based on the highly abundant ASVs (≥ 0.1% relative abundance)

**Table 2:** Pairwise comparisons of microbial composition within habitats

	Pairwise habitats	Observed statistic	Free stepdown adj. ***p***-value
**Caterpillars**	dunes - riverine grass.	354.2	**0.001**
	nat. grass. - riverine grass.	324.8	**0.006**
	dunes - nat. grass.	302.3	**0.007**
**Soil**	dunes - riverine grass.	16,252	**0.001**
	nat. grass. - riverine grass.	37,025	**0.001**
	dunes - nat. grass.	13,038	**0.001**

A negative binomial GLM was used. Multivariate test statistics were calculated using the log-likelihood ratio with 999 iterations via PIT-trap resampling. Significant results (*p-value* < 0.05) are in bold

Pairwise comparison of bacterial community composition of caterpillars and soil samples in within the three habitats based on the highly abundant ASVs (≥ 0.1% relative abundance)

### Shared microbiome between caterpillars and soil

Any caterpillar and soil sample shared a total of 989 ASVs regardless of sampling locality or habitat, of which 69% (683 ASVs) overlapped in the same habitat, and 64–84% of these were unique to one of the habitats (Fig. 2). The ASVs exclusively found in caterpillars and soils of coastal dunes, natural grasslands and riverine grasslands represented 12.8, 19 and 9.6% of the caterpillar’s total microbiomes, respectively. In total, 18 ASVs were common to caterpillar and soil samples across the three habitats representing 2.3% of the mean relative abundance of the caterpillar microbiomes (SI Table S5). Most of the shared ASVs were rare and occurred in low abundances in caterpillars but could reach high prevalence and abundance in the soil samples (Fig. 4). Yet, one ASV identified as Oligoflexales occurred consistently in the three habitats in 70% of all caterpillar samples; two ASVs identified as *Nitrobacter* and *Bradyrhizobium* were found in both caterpillar and soil samples in coastal dunes and natural grasslands, but only in caterpillar samples in riverine grasslands. These two absent taxa in riverine grassland soil samples could be due to a failure in detecting them in the soil samples, as we have shown that rarefaction curves did not reach a plateau (SI Figure S3).

## Discussion

Our study reveals that the monophagous caterpillars of the cinnabar moth *T. jacobaeae* collected from the field present a highly consistent set of a few dominant bacteria across three different habitats located more than one hundred kilometers apart. However, we also found that local microbial communities present in the soil may contribute to the variable caterpillar microbiomes, but that these microbes do not belong to the dominant taxa present in the caterpillar.

The high consistency of the most abundant bacterial ASVs detected in all caterpillars across habitats, irrespective of their size (Fig. 3), and the high number but lower abundance of specific taxa characteristic to each habitat (Fig. 2) suggest a relatively stable internal bacterial community composition of particular key taxa, likely influenced by the caterpillar’s physiology or adaptation to exclusive feeding on ragwort plants and phytochemicals such as pyrrolizidine alkaloids. These stable bacterial communities were observed irrespective of the variable percentage of chloroplast and mitochondria reads per sample (Figure S2) that were discarded, which could be indicative of the time since caterpillars last fed. The presence of specific microbial communities in the guts of insects combined with acquiring them through the environment in each generation has been recorded in other plant-feeding insects such as *Nezara viridula* (L.) (Heteroptera: Pentatomidae) [38] and *Riptortus clavatus* (Thunberg) (Heteroptera: Alydidae) [39], suggesting that environmental transmission is compatible with high specificity of microbial communities. The persistent presence and dominance of specific microbiota in caterpillars is unexpected due to the inhospitable environment of their midgut, with an unusual alkaline pH unfavorable to microbial growth [7]. We used whole caterpillars in our study that were surface-sterilized [19], thus the precise location of the bacteria inside the caterpillars is not known. It is possible that they originate from their midgut, hindgut or even salivary glands, and further research is required to clarify their origin. It is possible though that *T. jacobaeae* caterpillars rely on the bacterial ASVs found in this study as a fixed set of beneficial bacteria that could be considered to form a core microbiome for these caterpillars. Alternatively, it is also possible that the dominant ASVs were obtained when consuming the host plant tissues. Since these ASVs were detected in all samples, this would suggest that these bacteria are commonly associated to the host plant *J. vulgaris*. Which of these potential explanations is true, and what role these bacterial species play in insects and host plants remains to be investigated. Overall, the dependence level of monophagous and polyphagous caterpillars on their microbiomes is not well understood. Our study indicates that caterpillars of *T. jacobaeae* may represent an exception to the symbiont-independent feeding strategy found in most species in Lepidoptera [8], and further investigations are urged in this direction.

The functional relevance of the dominant bacteria found in *T. jacobaeae* (Fig. 3) is still unknown. *T. jacobaeae* feeds exclusively on ragwort plants containing pyrrolozidine alkaloids, and this specialization likely results from a physiological adaptation to its nutritional and allelochemical content [40], and the alkaloids can be used as defense [41–43], enabling them to exploit its food resource and avoiding competition and predation [16]. Gut bacteria of insects that specialize on toxic plants may play a crucial role in conferring the ability to digest or detoxify their food sources [2]. If the presence of these bacteria represents an evolutionary advantage for maintaining a consistent microbiome for *T. jacobaeae* to feed on ragwort plants that contain pyrrolozidine alkaloids avoiding its toxicity, they could be obtained via vertical transmission. *Burkholderia* spp. have been characterized as symbionts in e.g. broad-headed bugs [44], but there is barely any evidence for vertically transmitted caterpillar symbionts (but see [45, 46]). Alternatively, *T. jacobaeae* could obtain these bacteria from the ragwort plants and reflect their microbial communities, as Burkholderiaceae, and specifically taxa from the complex that includes *Paraburkholderia,* are often detected in plant leaves and considered plant-beneficial bacteria ([47] and references therein). Finally, the caterpillars could act as a vector of transmission of these bacteria between plants, as it has been shown in honeybees that carry the mutualist *Streptomyces* bacteria and transfer them between plants to protect them both from pathogens [48]. Yet, further studies are required to fully understand the function and the origin of these dominant bacteria.

*Ralstonia*, which comprises important plant pathogens [49] is the most dominant bacteria in *T. jacobaeae* caterpillars, representing a mean 53% of their microbiomes. Unfortunately, with the 16S rRNA gene fragment we sequenced it is not possible to identify the detected *Ralstonia* to species level preventing us to understand the biological function within the caterpillars. Yet, *Ralstonia* has been found to be commonly present in the guts of other insects, such as planthoppers and yellow ladybirds, and for the lepidopteran moth species, *Spodoptera littoralis* this bacteria has been detected at different developmental stages, including inside insect egg masses suggesting that this species may be transmitted from adults to offspring [1]. Furthermore, *Pseudomonas* species include common insect pathogens found in their guts [50]. Therefore, not all the dominant bacteria found in the caterpillars are expected to establish a unique relationship with the host. With our work though, we suggest that the soil is a less likely source for these dominant taxa, since none of the Burkholderiaceae or the *Pseudomonas* sp. found in *T. jacobaeae* were found in any soil sample of any habitat. Understanding the origin of these dominant bacteria, and whether they play a role in allowing the caterpillar to withstand the toxic compounds of the plant or participate in other functions within the caterpillar’s physiology requires further attention.

Our observation from this field survey of the microbiome of a specialist caterpillar in different habitats that the majority of bacterial ASVs found in *T. jacobaeae* were rare (i.e. found in less than three samples), provides unique information about the microbiomes of field collected caterpillars. However, the results resemble previous studies reporting that most bacteria were detected in individual samples in insects [46] and also mostly in environmental samples [51]. Nonetheless, the main phyla comprising the bacterial communities of *T. jacobaeae* were previously found to be characteristic of other caterpillar guts, with Proteobacteria being dominant in most insect species, even though different species seem to harbor characteristic proportions of each phylum [10, 52]. This supports the idea that despite being diffuse, bacterial assemblages in caterpillar guts are not random, suggesting some evolutionary constraint in the associations with bacteria of the different lineages of insects [2, 10]. Furthermore, only 5% of the total bacterial ASVs representing 86.2% of their total microbiomes (Fig. 2a) were detected in caterpillars across the three habitats, while 64–78% of the total ASVs were present exclusively in one habitat, reflecting that the community structure was influenced by the habitat (Fig. 4; Tables 1 and 2) and likely contributing to its transient character [8]. In addition, to this high variability detected in each habitat, bacteria diversity could be population driven since the adult moths can disperse over relatively large distances when laying their eggs, and nearby plants may not harbor eggs from the same moth mother, resulting in a less pronounced effect in closer localities, as observed by the lack of differences between the localities (SI Table S4).

**Fig. 5:**
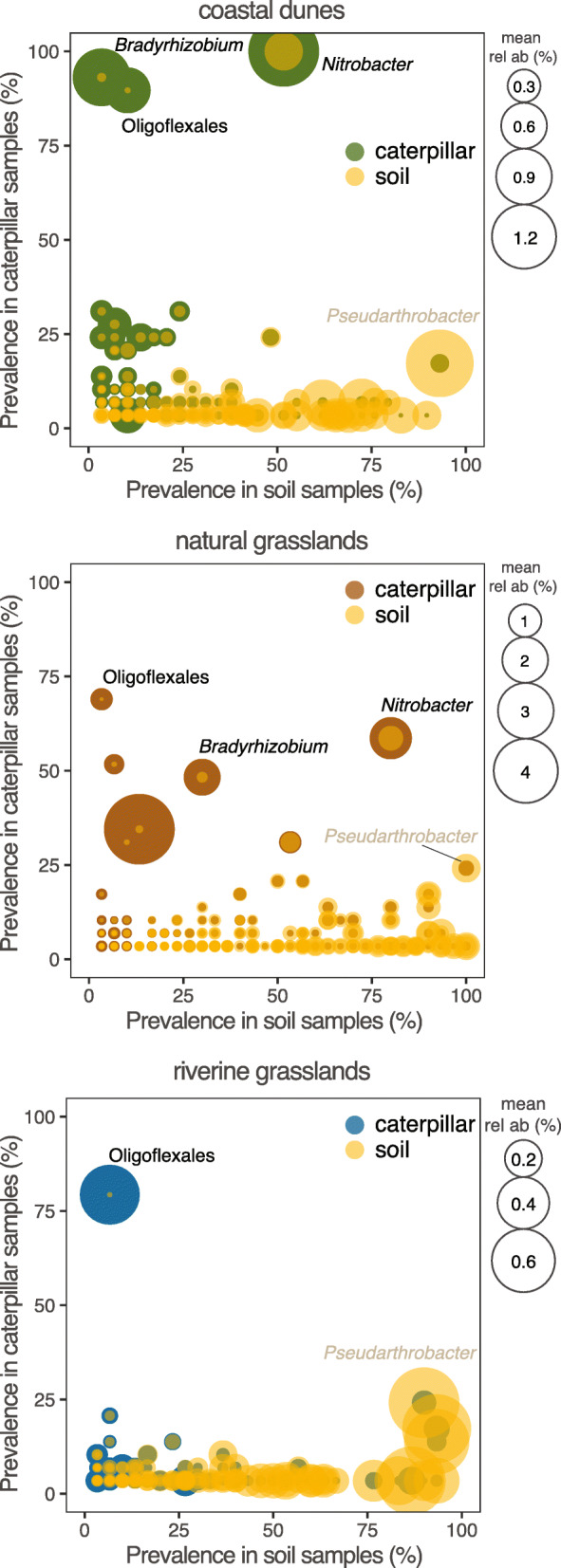
Shared microbiomes between caterpillar and soil samples in the three habitats. There were 221, 390 and 176 ASVs in common between caterpillar and soil samples in coastal dunes, natural grasslands and riverine grasslands, respectively. Most prevalent taxa in caterpillars are depicted. The same ASV identified as Oligoflexales occurred in the three habitats. ASVs identified as *Nitrobacter* and *Bradyrhizobium* were found in both caterpillar and soil samples in coastal dunes and natural grasslands, and only in caterpillar samples in riverine grasslands. *Pseudarthrobacter* was the most prevalent ASV in soil in the three habitats and occurred in roughly 25% of all caterpillars

In natural grasslands, almost 20% of the caterpillar microbiome was shared with the soil, while in the other two habitats this was roughly 10% (Fig. 2b). Most of the shared bacteria with the soil (12% of the microbiome) were unique to each habitat, an additional 11% were found in common in at least two of the habitats, and only 2.3% were shared between the three habitats, which is in agreement with previous works indicating that environment [53] and soil [13] shape caterpillars microbiomes. Even though these caterpillars feed exclusively on ragwort plants, they will travel between host plants after exhaustion of a plant and by that move over the soil and hence come in contact with the soil microbiome. It is also possible that the caterpillars actively move from the plant to the soil to acquire these bacteria (e.g. [47]) or that the bacteria are transferred from the soil via the host plant e.g. via the phyllosphere. However, previous work on other insects suggests that the latter may not be the case as caterpillars can have limited overlap between leaf microbiomes and their own [13]. It is not clear whether these bacteria shared with the soil (Fig. 5) act as symbionts of the caterpillars or represent widespread soil bacteria across habitats that are occasionally taken from the soil. Due to the specificity with each habitat, we suggest that these caterpillars take up part of their transient microbiomes from the soil. Such variability in bacterial communities could be beneficial if the ability to harbor a dynamic microbiome increases the chances to adapt to changing environmental conditions, or if those specific taxa found in the various environments provide redundant functional benefits to the caterpillars. For example, in termites, low abundant bacteria that often do not belong to their core microbiome have been reported to drive bacterial community composition upon dietary change [54].

## Conclusions

Caterpillars generally exhibit transient microbiomes but most of our knowledge is based on laboratory reared insects. In this study, with field collected caterpillars we found evidence for a low diverse stable microbiome, but also that the majority of individual bacterial ASVs associated with *T. jacobaeae* are rare and a quarter of their microbiomes reflected the soil microbial communities from the local habitat where they were collected. In fact, 86.2% of their microbiome was represented by only 5% of the total ASVs. Interestingly, one ASV affiliated with the genus *Ralstonia* was detected in all caterpillar samples representing 53% of the total caterpillars’ microbiome. Whether the most prevalent ASVs originate from the host plant or compose the functional core microbiome of this specialized caterpillar requires further studies. Our findings demonstrate that caterpillars in the field can have highly stable microbiomes, and simultaneously harbor a variable microbiome that reflects local soil bacterial communities.

## Supplementary information

**Additional file 1.**

## Data Availability

The dataset generated during the current study is available in the European Nucleotide Archive under the accession number PRJEB40063. The metadata file with sample details is deposited in Dryad (doi:10.5061/dryad.8cz8w9gnc). The R code used for the analyses presented in this manuscript is available upon request.
