# Supplementary material for: Microbiomes of a specialist caterpillar are consistent across different habitats but also resemble the local soil microbial communities
Source: Anim Microbiome. 2020 Oct 7;2:37. doi: 10.1186/s42523-020-00055-3 (PMC7807420; doi:10.1186/s42523-020-00055-3)
Supplement: Supplementary file 1 — Additional file 1. [file 42523_2020_55_MOESM1_ESM.docx]

**Supplementary Information**

**Microbiomes of a specialist caterpillar are consistent across different habitats but also resemble the local soil microbial communities**

Sofia I.F. Gomes^*^, Anna M. Kielak^*^, S. Emilia Hannula, Robin Heinen, Renske Jongen, Ivor Keesmaat, Jonathan R. De Long, T. Martijn Bezemer

**Tables**

**Table S1**: Diversity indices (observed and estimated) and average fresh weight of caterpillars.

**Table S2**: Diversity indices (observed and estimated) of soil samples.

**Table S3**: Most dominant bacterial ASV members of caterpillar’s microbiome. Prevalence in the total 87 caterpillar samples and its mean relative abundance per habitat is presented. See Figure 2 for prevalence and relative abundance per habitat.

**Table S4**: Pairwise comparison of bacterial community composition of caterpillars and soil samples in within the nine localities regions, using the rarefied dataset.

**Table S5**: Taxonomy and distribution of the 18 shared bacterial ASVs between caterpillar and soil samples across the three regions. Prevalence (Prev) and % relative abundance (Relab) of the common ASVs are depicted for caterpillar samples within each habitat.

**Figures**

**Figure S1:** Sampling sites within the nine localities within the three regions in the Netherlands.

**Figure S2:** Percentage of chloroplast and mitochondria reads in caterpillars per locality (left panel) and per habitat (right panel). Kruskal-Wallis tests show no significant differences between localities (*chi-squared* = 14.612, *df* = 8, *p* = 0.067) or regions (*chi-squared* = 0.372, *df* = 2, *p* = 0.830).

**Figure S3:** Average caterpillars’ weight used for DNA extractions from each sampling locality.

**Figure S4:** Sample-size rarefaction (solid lines) and extrapolation (dashed lines) sampling curves with confidence intervals of caterpillar and soil samples per location (9 samples per location). Caterpillar samples were resampled to 3,849 reads and soil samples to 13,731 reads.

**Figure S5:** Spearman correlations between caterpillars’ fresh weight (g) and bacterial alpha diversity indices. Spearman’s rank correlation (*rho*) and *p-values* are depicted.

**Figure S6:** Microbiome community composition of caterpillars represented by Principal Coordinates Analysis as in Figure 4 with samples highlighted according to caterpillar weight. Bacteria community composition is not explained by caterpillar weight (multivariate GLM: *df* = 85, *deviance* = 4327, *p* = 0.059).

**Tables**

**Table S1**: Diversity indices (observed and estimated) and average fresh weight (g) of caterpillars.

**Table S2**: Diversity indices (observed and estimated) of soil samples.

**Table S3**: Most dominant bacterial ASV members of the caterpillar microbiome. Prevalence in the total 87 caterpillar samples that were analyzed and its mean relative abundance per habitat is presented. See Figure 2 for prevalence and relative abundance per habitat.

**Table S4**: Pairwise comparisons of bacterial community composition of caterpillars and soil samples between each location, using the rarefied dataset.

**Table S5**: Taxonomy and distribution of the 18 shared bacterial ASVs between caterpillar and soil samples across the three regions. Prevalence (Prev) and % relative abundance (Relab) of the common ASVs are depicted for caterpillar samples within each habitat.

**Figures**

**Figure S1:** Sampling sites within the nine localities within the three regions in the Netherlands.


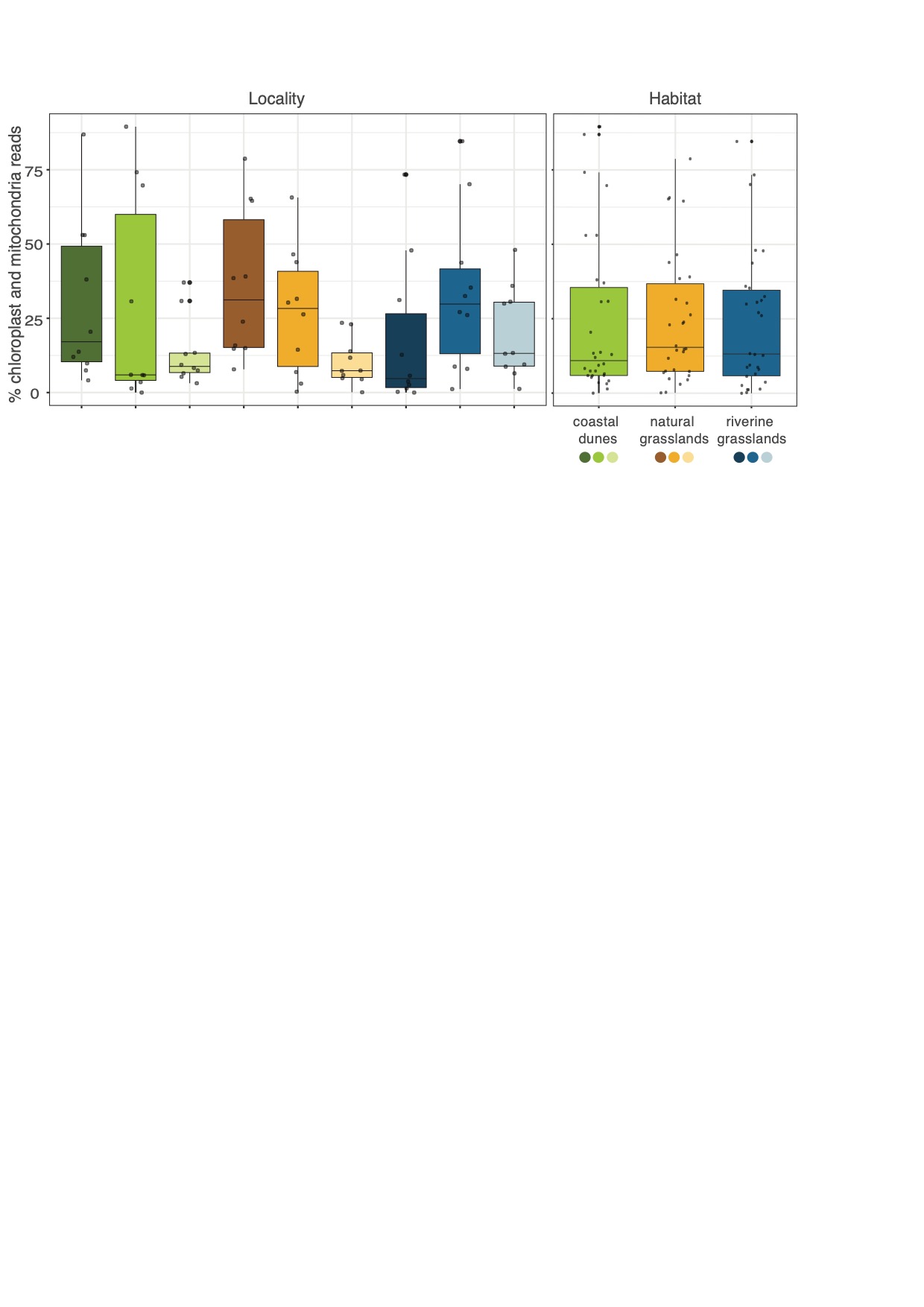


**Figure S2:** Percentage of chloroplast and mitochondria reads in caterpillars per locality (left panel) and per habitat (right panel). Kruskal-Wallis tests show no significant differences between localities (*chi-squared* = 14.612, *df* = 8, *p* = 0.067) or regions (*chi-squared* = 0.372, *df* = 2, *p* = 0.830).


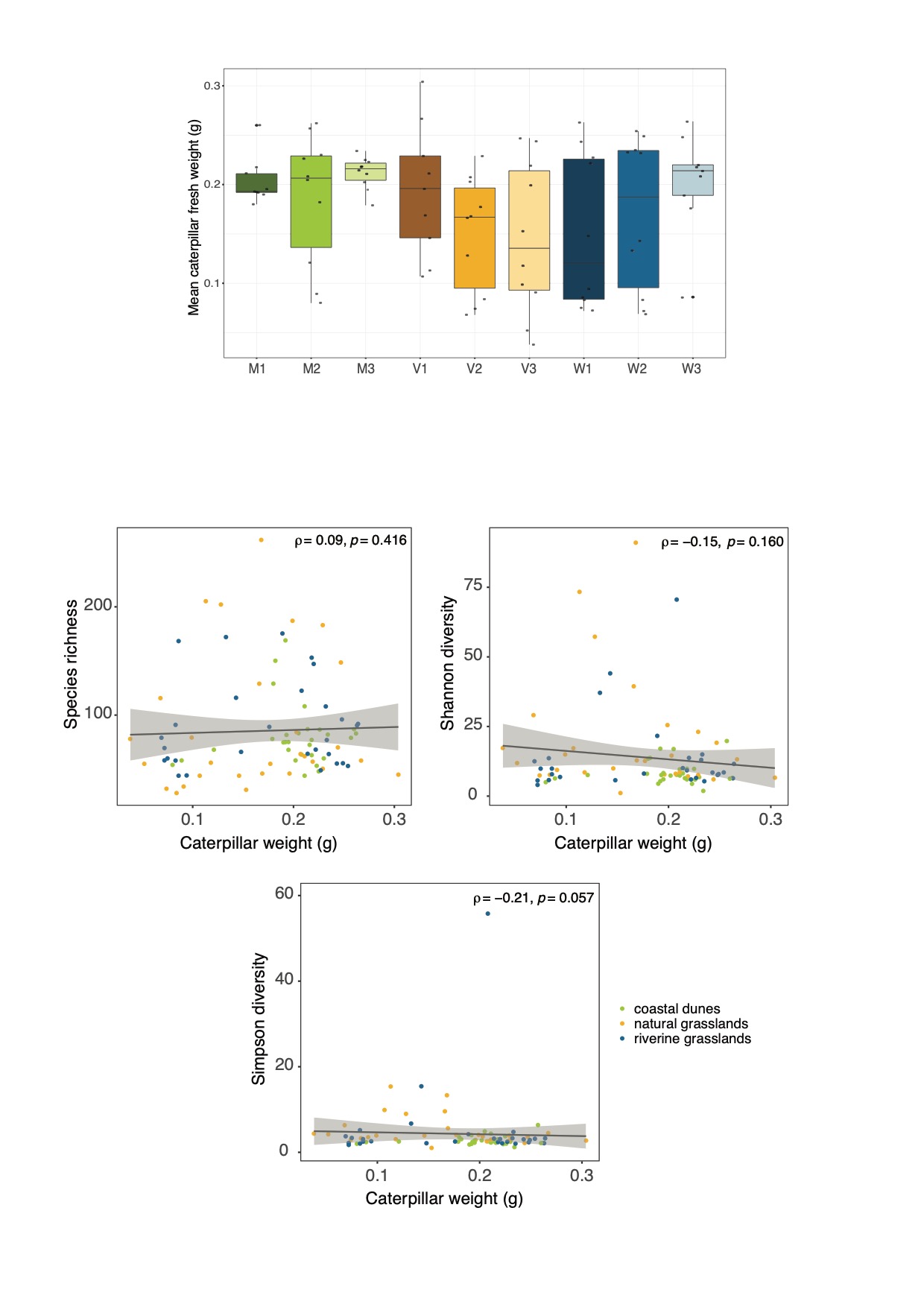


**Figure S3:** Average weight of the caterpillars used for DNA extractions from each sampling locality.

**
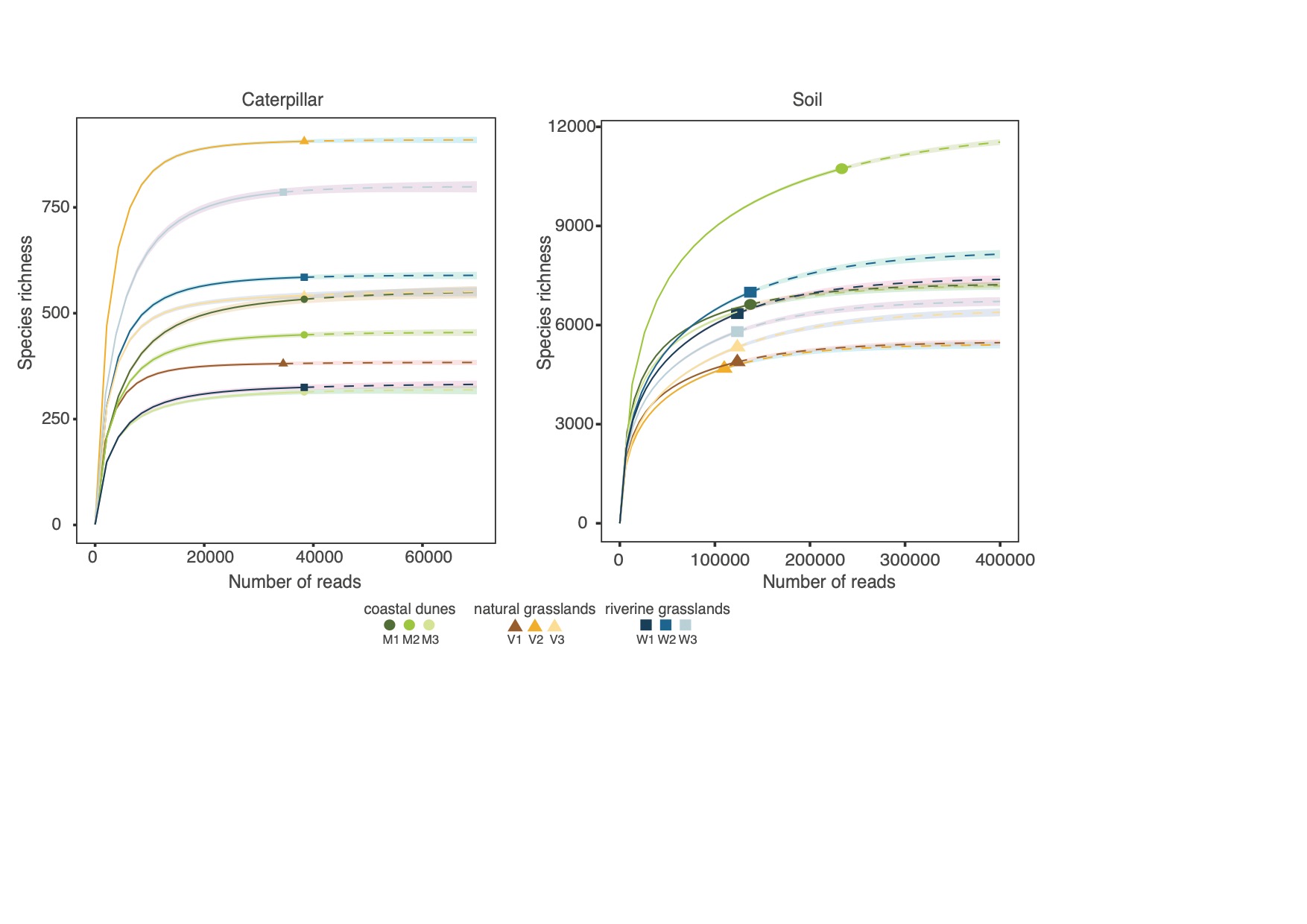
**

**Figure S4:** Sample-size rarefaction (solid lines) and extrapolation (dashed lines) sampling curves with confidence intervals of caterpillar and soil samples per location (9 samples per location). Caterpillar samples were resampled to 3,849 reads and soil samples to 13,731 reads.


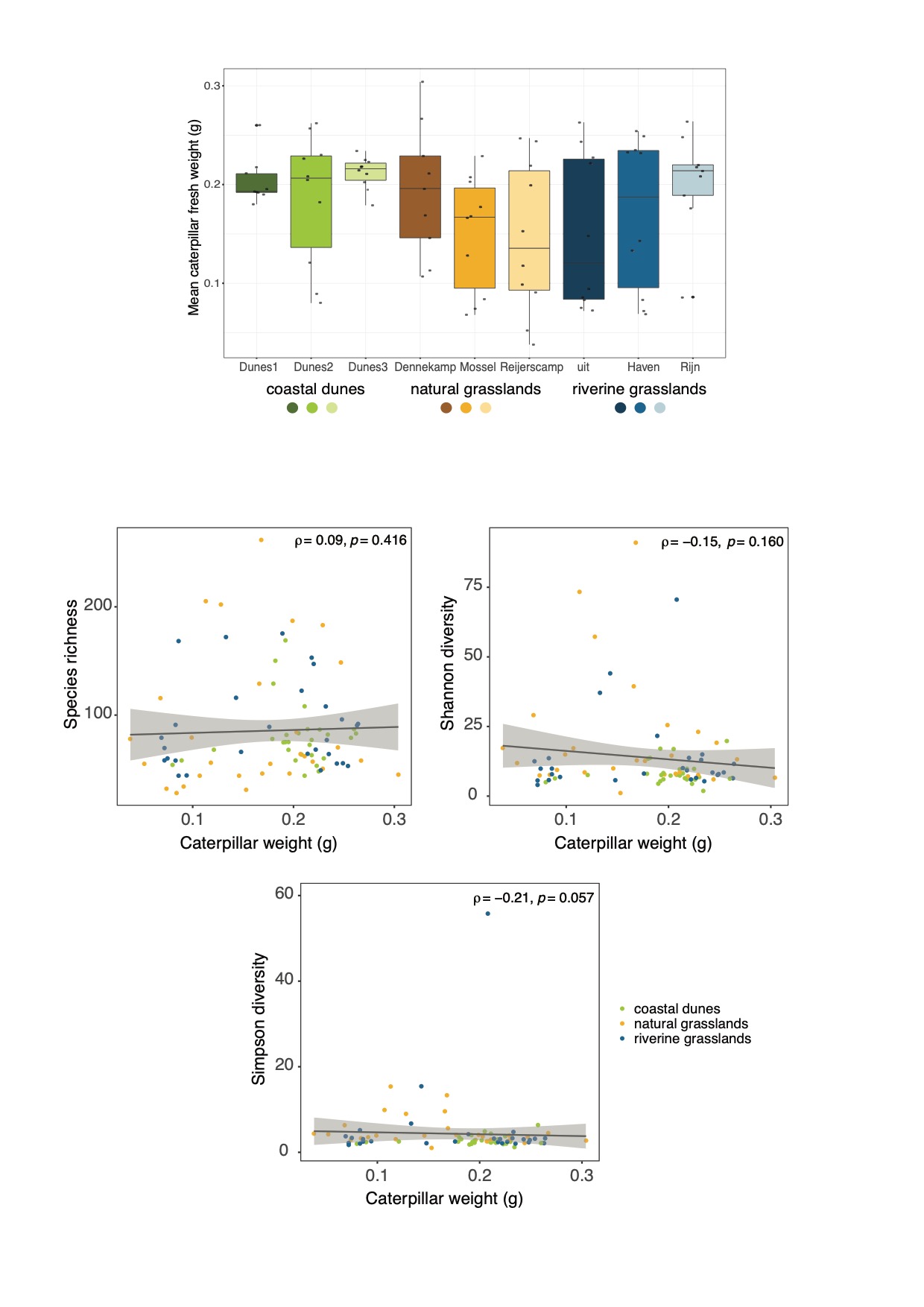


**Figure S5:** Spearman correlations between mean caterpillar fresh weight per plant (g) and bacterial alpha diversity indices. Spearman’s rank correlation (*rho*) and *p-values* are depicted.

**Figure S6:** Microbiome community composition of caterpillars represented by Principal Coordinates Analysis as in Figure 4 with samples highlighted according to caterpillar weight. Bacteria community composition is not explained by caterpillar weight (multivariate GLM: *df* = 85, *deviance* = 4327, *p* = 0.059).
